# Cross-cultural adaptation of the eHealth Literacy Scale for Brazilian adolescents

**DOI:** 10.1590/1807-3107bor-2024.vol38.0094

**Published:** 2024-09-30

**Authors:** Mariane Carolina Faria BARBOSA, Ana Luiza Peres BALDIOTTI, Maria Luisa Leandro de Souza DIAS, Ana Flávia GRANVILLE-GARCIA, Saul Martins PAIVA, Fernanda de Morais FERREIRA

**Affiliations:** (a) Universidade Federal de Minas Gerais – UFMG, School of Dentistry, Departament of Pediatric Dentistry, Belo Horizonte, MG, Brazil.; (b) Universidade Estadual da Paraíba – UEPB, Postgraduate Program in Dentistry, Campina Grande, PB, Brazil.

**Keywords:** Health Literacy, Cross-Cultural Comparison, Internet Access, Telemedicine, Adolescent

## Abstract

The aim of this study was to undertake a cross-cultural adaptation of the eHealth Literacy Scale (eHEALS) instrument to measure digital health literacy of Brazilian adolescents. eHEALS is a scale consisting of 8 items that measure self-perception related to the consumption of electronic health information. This is a methodological study of cross-cultural adaptation, conducted out from February 2022 to June 2022. The following steps were carried out: a) assessment and adequacy of cultural equivalence by a committee of experts; b) back-translation; c) synthesis of back-translations; d) cognitive testing with 42 Brazilian adolescents, using cognitive interviews with probing questions. All items that were difficult to understand were adapted to the (language) context of Brazilian adolescents. Cronbach’s alpha coefficient for eHEALS-BrA was 0.81 and, if one of the items were excluded from the instrument, it ranged from 0.75-0.81. This version of the eHEALS proved to be culturally well-adapted to the context of Brazilian adolescents, and has the potential to measure digital health literacy in this population after having its validation confirmed through psychometric analyses.

## Introduction

Health literacy (HL) is a construct that includes the ability to obtain, process, interpret and apply basic health information to make appropriate decisions.^1^ It is considered an important social determinant of health since limited levels of HL are generally found in vulnerable socioeconomic groups that have barriers to accessing and understanding health care.^2,3^

In recent years, there has been an increase in the population’s access to information and communication technologies (ICTs).^4,5^ In Brazil, approximately 82.7% of households have an internet connection and, in relation to adolescents, 90.2% are connected.^6^ It is noteworthy that most users obtain health knowledge by means of digital platforms, such as search engines and social media.^4,7,8^ However, much of this information available online does not pass the quality check,^8,9^ and this can lead to the consumption of false information, consequently, misinforming individuals and promoting beliefs that are harmful to health.^10,11^

Therefore, for the proper consumption of digital health content, individuals must have good levels of Digital Health Literacy (DHL); that is, they must be able to search, find, understand and evaluate health information in electronic sources, and apply the knowledge acquired to approach or solve a health problem.^12^ Worth emphasizing is the fact that socially vulnerable individuals are equally likely to be faced with challenges by the HL and the DHL.^5,10^

With these aspects in mind, the eHealth Literacy Scale (eHEALS) was developed. This tool is a self-administered instrument to assess the level of DHL.^12^ The scale consists of eight items ^12^ and to the best of our knowledge, it has been validated for adults^9^ and university students^3^ in Brazil, when it presented average values of 25.1 (± 8.1) and 28.0 (± 5.53) points, respectively. Despite the validation with Brazilian undergraduate students, which included a portion of the age range of adolescents, the mean age of the undergraduate was 21.2 (± 3.7) years.^3^

Adolescence, a time of transition from childhood to adulthood, is marked by dynamic neurobiological and behavioral changes.^13,14^ Adolescents have unique characteristics and are more emotional and impulsive. Moreover, they are still developing their cognitive processes, the decision-making skills, and are susceptible to social influences. Adolescents use the internet with great frequency and intensity, often looking for health information online.^14-16^ In the US, a study showed that 84% of adolescents have used the internet to access health information.^17^ In view of the absence of an instrument to measure DHL for Brazilian adolescents and the need to teach them about e-health and DHL as early as possible, the aim of the present study was to undertake a cross-cultural adaptation of the eHealth Literacy Scale (eHEALS) for Brazilian adolescents.

## Methods

### Description of the instrument

The eHealth Literacy Scale is the first self-report instrument that has been developed to meet the need to measure digital health literacy for a wide range of populations and contexts. It was developed in the English language and in the cultural context of Canada, based on a model of six eHealth skills or types of literacy: traditional literacy, health literacy, information literacy, scientific literacy, digital literacy and computer literacy.^12^

The instrument is composed of eight items that are evaluated using a 5-point Likert scale, ranging from 1 (completely disagree) to 5 (completely agree), according to the respondents’ perception. The total score can vary from 8 to 40 points, with higher scores indicating a higher level of digital literacy in self-perceived and self-reported health.^12^

### Study design and ethical aspects

A methodological study of cross-cultural adaptation was conducted with individuals aged 13 to 19, enrolled in three public schools in Belo Horizonte, Brazil. The cross-cultural adaptation process took place in the period from February to June 2022.

Prior to its implementation, authorization was requested from the authors of the original instrument ^12^ and approval was obtained from the Ethics Committee for Research with Human Beings of the Federal University of Minas Gerais (#CAAE:51689627.1.0000.5149). All guardians of adolescents or those over 18 years of age signed the Term of Free and Informed Consent and the participants confirmed their participation by signing the Term of Free and Informed Assent.

### Cross-cultural adaptation

The COSMIN Study Design checklist was used with reference to the process of cross-cultural adaptation.^18^ In addition, a universalist approach was adopted to performing the translation and cross-cultural adaptation.^19,20^

The Brazilian Portuguese version of the eHEALS instrument, which has been cross-culturally adapted and validated for Brazilian university students, was used.^3^ To achieve the proposed objectives, the following steps were taken: a) assessment and adequacy of cultural equivalence by a committee of experts; b) back-translation; c) synthesis of back-translations; d) cognitive testing with Brazilian adolescents, using cognitive interviews with probing questions (Figure).

**Figure:**
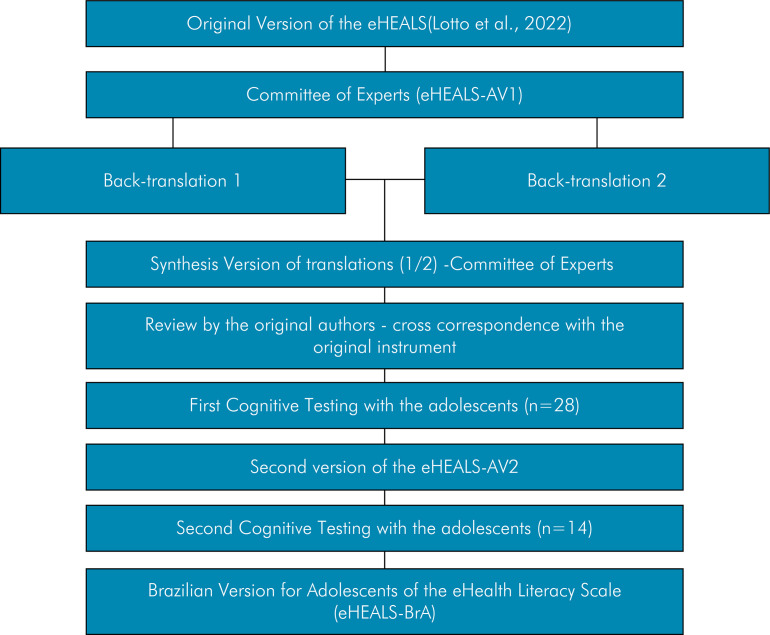
Flowchart of the adaptation process of eHEALS-BrA to Brazilian Portuguese.

### Committee of Experts

The Brazilian version of the eHEALS instrument validated for university students^3^ was submitted to three Brazilian experts for appreciation. These experts are native speakers of Brazilian Portuguese, who work as higher education professors and have training in the area of health and expertise in Health Literacy. For this stage, two meetings were held with the researchers and committee members, who had previously received the original instrument and the Brazilian version for university students.

This step was performed to assess equivalence with the original instrument, and to evaluate the clarity of language for the age group of 13 to 19 years. The experts could suggest changes and formulate expressions that they deemed to be more relevant for each of the items. This process originated the initial version of the eHEALS instrument adapted for use in Brazilian adolescents (eHEALS-AV1).

### Back-translation

At this stage, two independent translators, native speakers of English, who have linguistic and cultural mastery of English and Portuguese and who were blind to the original version of the instrument and the objectives of the study, performed the back-translation of the eHEALS-AV1 version into English.

### Synthesis of back-translations

A committee of three Brazilian experts trained in the field of health and fluent in English made a new assessment and comparison between the back-translated versions and the original instruments, for the purpose of obtaining agreement on the back-translated synthesis version of the instrument in English.

The synthesis version was sent to the original authors for evaluation of operational equivalence. The authors agreed with the back-translated version and, therefore, it was not necessary to make adaptations to the eHEALS-AV1 version at this time.

### Cognitive testing

A sample of 42 adolescents aged between 13 and 19 years participated in this stage. Adolescents were eligible when they met the following requirements: they had to be native speakers of Brazilian Portuguese, have regular access to the internet and did not have any self-reported or reported vision, hearing or cognitive impairments that would prevent their participation in the study.

In this step, volunteers were selected by means of distribution by age and sex, in addition to having characteristics of access to internet similar to those of the Brazilian population.^6^ A representative sample of the target population of the instrument was selected, with the aim of obtaining a version of eHEALS that was adapted to the reality of Brazilian adolescents.

The first phase, the first pre-test, with the eHEALS-AV1 version, was performed with a convenience sample of 28 adolescents, composed of 14 boys and 14 girls. The aim of the test was to evaluate the applicability of the instrument, verify the understanding of the items, emotional reaction and acceptability of the instrument layout. Adolescents were encouraged to point out difficulties with clarity and suggest synonyms for terms or words that were difficult to understand.

The questionnaire was applied in a standardized manner and later an individual cognitive interview was held, using probing questions and conducted by one of the researchers (M.C.F.B.). A committee of three specialists jointly evaluated all the adolescents’ doubts, suggestions and notes during the first pre-test, and made adjustments that did not change the conceptual meaning of the original item.

A second version (eHEALS-AV2) was obtained, which was presented to 14 new volunteers, 6 women and 8 men. In this step (second pre-test), the instrument was applied and a cognitive interview with probing questions was held. It was not necessary to make new adjustments, therefore, the Brazilian Version for Adolescents of the eHealth Literacy Scale (eHEALS-BrA) was obtained.

### Statistical analysis

Descriptive data analysis was performed using the SPSS Statistics 21.0 program (SPSS Inc., Chicago, USA). Cronbach’s alpha was used to assess the reliability of the final version of the instrument through internal consistency α > 0.61).^21^

## Results

Table 1 presents the result of the cross-cultural adaptation of eHEALS for adolescents in the context of Brazil. The first column presents the items of the original version ^12^ of the instrument in English and the second column presents the final version in Portuguese validated for university students.^3^ Column three presents the final version in Brazilian Portuguese for adolescents after the changes made by the expert committee, based on analysis of the translations and back-translations, notes and suggestions of the adolescents during the pre-test and on the clarifications of the authors of the original instrument.

**Table 1:** Original Version for eHealth Literacy Scale and Brazilian Version for Adolescents of the eHealth Literacy Scale (eHEALS-BrA).

Itens	Original Version	Brazilian Version for Adolescents of the eHealth Literacy Scale (eHEALS-BrA).
	eHealth Literacy Scale items^11^	
Statement	I would like to ask you for your opinion and about your experience using the Internet for health information. For each statement, tell me which response best reflects your opinion and experience right now.	Para as questões abaixo, eu gostaria de saber sua opinião e sua experiência no uso da Internet para buscar informações sobre saúde. Não existem respostas certas ou erradas. Para cada afirmação, marque a resposta que melhor reflete sua opinião e sua experiência neste momento.
		Considere “Recursos de saúde” como todas as formas de usar a internet relacionada a saúde.
Answer	1) Strongly Disagree	a) Discordo totalmente
	2) Disagree	b) Discordo em parte
	3) Undecided	c) Não tenho certeza
	4) Agree	d) Concordo em parte
	5) Strongly Agree	e) Concordo totalmente
1.	I know how to find helpful health resources on the Internet	Eu sei como encontrar informações/recursos úteis de saúde na Internet.
2.	I know how to use the Internet to answer my questions about health	Eu sei como usar a Internet para responder as minhas dúvidas sobre saúde.
3.	I know what health resources are available on the Internet	Eu sei quais os recursos de saúde que estão disponíveis na Internet.
4.	I know where to find helpful health resources on the Internet	Eu sei onde encontrar informações/recursos úteis de saúde na Internet.
5.	I know how to use the health information I find on the Internet to help me	Eu sei como usar as informações de saúde que encontro na internet para me ajudar.
6.	I have the skills I need to evaluate the health resources I find on the Internet	Eu tenho as habilidades necessárias para avaliar as informações/recursos de saúde que encontro na internet.
7.	I can tell high quality health resources from low quality health resources on the Internet	Eu consigo diferenciar as informações/recursos de saúde de alta qualidade das de baixa qualidade na internet.
8.	I feel confident in using information from the Internet to make health decisions	Eu me sinto confiante em usar as informações da Internet para tomar decisões sobre saúde.

During the expert committee stage, the equivalence of items, semantics and conceptual expressions was evaluated. There was a need to include an explanation for the term “Health resources” in the statement of the instrument with the aim of making it easier for the age group to understand it. Furthermore, it was decided to change three of the five response options on the Likert scale, Disagree/Undecided/Agree for I partially Disagree/Not sure/Partly Agree. Other proposals for improvement made by the specialists were incorporated into the instrument, such as marking some words in the items in bold, similar to the original instrument, and the inclusion of the term “Information” before the expression “Health resources”.

During the back-translation stage, we found a level of agreement equal to 64.33% between the two back-translated versions. All of the 8 items of the two back-translations were considered little changed, apart from this, back-translation 1 was declared to be more adequate in terms of correspondence with the original version. Disagreements were resolved by consensus in the committee of three experts who developed the back-translated synthesis version.

During the pre-test, the eHEALS-BrA proved to be an easy-to-use instrument, with an average time of 3.06 minutes (1.5–10).required to fill in the form. Furthermore, relative to operational equivalence, the way of application and the layout of the instrument were found to be satisfactory and suitable for Brazilian adolescents.

In the first pre-test, the difficulty with understanding was identified only in the seventh item of the instrument: “I am capable of differentiating health information/ resources that are of high quality from those of low quality on the internet”. The item was adjusted to read “I can differentiate between high-quality and low-quality health information/resources on the internet”, which proved to be effective during the second pre-test stage.

Table 2 describes the characteristics of the adolescents who participated in the instrument pre-test, and of the eHEALS-BrA score (mean and standard deviation). These data were obtained by means of the complementary questionnaire on sociodemographic characterization and access to ICTs applied to the sample. The participants of this study were a total of 42 adolescents. with a mean age of 16.0 ± 2.0 (13–19) years and mean family income of R$ 2215.08 ± 1301.89. All participants had access to internet, used social networks and their main means of access to internet was via Smartphone (100%).

**Table 2:** Characteristics of the adolescents who participated in the instrument pre-test, and of the eHEALS-BrA score (mean and standard deviation) (n = 42).

Variable	n (%)	eHEALS-BrA Score
		Mean (SD)
Sex		
Female	20 (47.6)	24.6 ± 6.1
Male	22 (52.4)	27.0 ± 4.5
Age (years)		
≤ 15	19 (45.2)	24.7 ± 5.2
> 16–19	23 (54.8)	26.8 ± 5.4
Adolescents education level (years of study)		
< 8	35 (83.3)	
≥ 8	7 (16.7)	27.9 ± 6.8
Skin color (self-declared)		
Black	13 (31.0)	26.5 ± 6.3
White	13 (31.0)	25.4 ± 4.7
Brown	16 (38.0)	25.7 ± 5.5
Parents/guardians education level (years of study)		
< 8	16 (38.1)	27.1 ± 6.0
≥ 8	20 (47.6)	24.7 ± 5.4
Health of the adolescent (self-reported)		
Bad/Fair	8 (19.0)	25.9 ± 5.1
Good/Very good	34 (81.0)	25.6 ± 6.7
Oral health of the adolescent (self-reported)		
Bad/Fair	13 (31.0)	26.8 ± 5.6
Good/Very good	29 (69.0)	23.7 ± 4.4
Looking for health information on the internet		
Yes	24 (57.1)	26.2 ± 5.2
No	18 (42.9)	25.3 ± 5.7
Uses health-related smartphone app		
Yes	9 (21.4)	29.7 ± 3.2
No	33 (78.6)	24.8 ± 5.4
Do you have a Computer/Notebook/Tablet?		
Yes	18 (42.9)	27.0 ± 5.0
No	24 (57.1)	24.3 ± 5.6
Internet access frequency		
Every day	36 (85.7)	25.4 ± 5.4
Almost everyday	6 (14.3)	28.3 ± 4.6
Average time using the internet per day (years)		
0–5	16 (38.1)	28.0 ± 6.3
5–10	14 (33.3)	24.9 ± 5.1
≥ 10	12 (28.6)	24.0 ± 3.4
Did you follow guidelines/health tips from bloggers. digital influencers or people you follow on the social network?		
Yes	21 (50.0)	26.1 ± 4.9
No	21 (50.0)	25.5 ± 5.9
Do you look for information about a doctor/dentist on social networks before consulting?		
Yes	8 (19.0)	24.1 ± 3.1
No	34 (81.0)	26.2 ± 5.7
Performed self-medication based on information available on the internet		
Yes	10 (23.8)	27.3 ± 6.2
No	32 (76.2)	25.4 ± 5.1

The score stipulated by the original instrument was maintained by obtaining the sum of the values assigned to each item (1: strongly disagree; 2: disagree; 3: I’m not sure; 4: I agree and 5: I totally agree), with a total score ranging from 8 to 40 points. In the eHEALS-BrA pre-test, the participating adolescents obtained a mean score of 25.83 ± 5.3 (16–36) points.

With reference to internal consistency of the instrument and each of the items, the eHEALS-BrA pre-test data showed satisfactory Cronbach’s Alpha values that could be considered almost perfect (α = 0.81),^21^ in addition to not showing major changes when a question was removed. These results were among the values accepted by the literature^20,21^ and were close to the alpha of the original instrument (α = 0.88)^12^. When one of the items was excluded from the instrument, Cronbach’s alpha ranged from 0.75–0.81 (Table 3).

**Table 3:** Cronbach’s Alpha coefficient for scores on the eHEALS-BrA if the item was excluded from the instrument (n = 42).

Itens	Cronbach’s Alpha coefficient if the item was excluded from the instrument
1 - Eu sei como encontrar informações/recursos úteis de saúde na Internet.	0.79
2 - Eu sei como usar a Internet para responder as minhas dúvidas sobre saúde.	0.79
3 - Eu sei quais os recursos de saúde que estão disponíveis na Internet.	0.81
4 - Eu sei onde encontrar informações/recursos úteis de saúde na Internet.	0.76
5 - Eu sei como usar as informações de saúde que encontro na internet para me ajudar.	0.75
6 - Eu tenho as habilidades necessárias para avaliar as informações/recursos de saúde que encontro na internet.	0.78
7 - Eu consigo diferenciar as informações/recursos de saúde de alta qualidade das de baixa qualidade na internet.	0.81
8 - Eu me sinto confiante em usar as informações da Internet para tomar decisões sobre saúde.	0.80

## Discussion

This is the first study that has performed a cross-cultural adaptation of the eHeatlh Literacy Scale instrument to measure digital health literacy among adolescents in Brazil. eHEALS was the first instrument developed to measure DHL, in addition to having good psychometric properties.^12^ It was, however, developed in English and in the cultural context of Canada,^12^ but it has previously been cross-culturally adapted and validated for adolescents of other countries.^22-24^

In order to improve scientific knowledge in the health sciences, methodological standardization is necessary using instruments previously adapted for the culture and age group of the population of interest. This standardization improves the quality of studies, comparability of results and reduces divergences; therefore, it promotes a better theoretical basis for planning public policies and (guiding) clinical management.^1,19,20^ Therefore, in the present study, a cross-cultural adaptation of this DHL measurement tool was performed, with the aim of assessing adolescents in the national territory of Brazil (eHEALS-BrA).

To perform the cross-cultural adaptation, a universalist approach was adopted to developing the steps recommended.^19,20^ Semantic, idiomatic, conceptual and cultural discrepancies were corrected. The analysis of eHEALS by the expert committee made it possible to correct inaccuracies and adjust the items to the Brazilian Portuguese language, cultural context and age group of Brazilian adolescents. Furthermore, application of eHEALS in the pre-test stages made it possible to investigate the clarity, understanding and level of formality of the adapted instrument. The adjustments suggested by the adolescents were made, such as replacing words and formal phrases that were difficult to understand with colloquial expressions and phrases.

This work has the potential to contribute to the surveillance and understanding of the DHL level of adolescents in Brazil, as it provides an adapted instrument, the eHEALS-BrA, which is capable of measuring the level of digital health literacy in this population, providing comparable results at national and international level. Apart from having excellent characteristics, it is a short instrument that is easy and fast to apply. Therefore, the availability of this epidemiological research tool could contribute to the expansion of studies on this topic.

Recently, the World Health Organization stated that the infodemic was a threat to public health and suggested that countries implement plans to combat misinformation.^25,26^ Adolescents need to develop abilities in judging the reliability and scientific soundness of health claims and information. However, they may be deficient in critical appraisal skills and the use of eHealth, which can persist into higher education and into adulthood.^27,2 8^ Thus, knowledge of the level of digital health literacy could provide relevant information to support the planning of public educational strategies and the promotion of e-health in adolescence. Furthermore, when designing e-Health services, it could help health organizations to adopt an approach based on reducing DHL-related disparities in the population, to provide digital accessibility to health for everybody.

The eHealth Literacy Scale is one of the instruments most widely used for assessing this construct, due to its good characteristics such as ease of application, versatility in different cultural contexts and excellent psychometric properties.^12,29^ Nevertheless, it is worth noting that this instrument has limitations, as it assesses the individuals’ perception of health information in the digital environment, and does not assess the skills required for digital health literacy.

Moreover, our study has some limitations; it was conducted with a small sample collected in only three municipal schools, with the aim of meeting the proposal of a cross-cultural adaptation for adolescents. Future studies should conduct a psychometric evaluation of eHEALS-BrA in larger samples of the Brazilian population aged 13 to 19 years, contemplating the analysis of its dimensional structure, by performing test-retests.

## Conclusion

The eHEALS-BrA proved to be culturally well-adapted to the context of Brazilian adolescents, and has the potential to measure digital health literacy in this population after having its validation confirmed through psychometric analyses.

## References

[1] Quemelo PR, Milani D, Bento VF, Vieira ER, Zaia JE (2017). Health literacy: translation and validation of a research instrument on health promotion in Brazil. Cad Saude Publica.

[2] Berkman ND, Sheridan SL, Donahue KE, Halpern DJ, Viera A, Crotty K (2011). Health literacy interventions and outcomes: an updated systematic review. Evid Rep Technol Assess (Full Rep).

[3] Lotto M, Maschio KF, Silva KK, Ayala Aguirre PE, Cruvinel A, Cruvinel T. (2023). eHEALS as a predictive factor of digital health information seeking behavior among Brazilian undergraduate students. Health Promot Int.

[4] Lotto M, Aguirre PE, Strieder AP, Cruvinel AF, Cruvinel T (2019). Levels of toothache-related interests of Google and YouTube users from developed and developing countries over time. PeerJ.

[5] Smith B, Magnani JW (2019). New technologies, new disparities: the intersection of electronic health and digital health literacy. Int J Cardiol.

[6] Instituto Brasileiro de Geografia e Estatística (2019). Pesquisa Nacional por Amostra de Domicílios Contínua: acesso à Internet e à televisão e posse de telefone móvel celular para uso pessoal 2018.

[7] Graf I, Gerwing H, Hoefer K, Ehlebracht D, Christ H, Braumann B (2020). Social media and orthodontics: a mixed-methods analysis of orthodontic-related posts on Twitter and Instagram. Am J Orthod Dentofacial Orthop.

[8] Lotto M, Sá Menezes T, Zakir Hussain I, Tsao SF, Ahmad Butt Z, P Morita P (2022). Characterization of false or misleading fluoride content on Instagram: infodemiology study. J Med Internet Res.

[9] Mialhe FL, Moraes KL, Sampaio HA, Brasil VV, Vila VD, Soares GH (2021). Evaluating the psychometric properties of the eHealth Literacy Scale in Brazilian adults. Rev Bras Enferm.

[10] Swire-Thompson B, Lazer D (2020). Public health and online misinformation: challenges and recommendations. Annu Rev Public Health.

[11] Galhardi CP, Freire NP, Fagundes MC, Minayo MC, Cunha IC (2022). Fake news and vaccine hesitancy in the COVID-19 pandemic in Brazil. Cien Saude Colet.

[12] Norman CD, Skinner HA (2006). eHEALS: the ehealth literacy scale. J Med Internet Res.

[13] Gaete V (2015). Adolescent psychosocial development. Rev Chil Pediatr.

[14] Freeman JL, Caldwell PH, Bennett PA, Scott KM (2018). How adolescents search for and appraise online health information: a systematic review. J Pediatr.

[15] Park E, Kwon M (2018). Health-related internet use by children and adolescents: systematic review. J Med Internet Res.

[16] Fleary SA, Joseph P, Pappagianopoulos JE (2018). Adolescent health literacy and health behaviors: a systematic review. J Adolesc.

[17] Wartella E, Rideout V, Montague H, Beaudoin-Ryan L, Lauricella A (2016). Teens, health and technology: a national survey. Media Commun.

[18] Mokkink LB, Terwee CB, Patrick DL, Alonso J, Stratford PW, Knol DL (2010). The COSMIN checklist for assessing the methodological quality of studies on measurement properties of health status measurement instruments: an international Delphi study. Qual Life Res.

[19] Paiva SM, Firmino RT, Abeu LG, Estrela C (2018). Metodologia científica: ciência, ensino, pesquisa.

[20] Beaton DE, Bombardier C, Guillemin F, Ferraz MB (2000). Guidelines for the process of cross-cultural adaptation of self-report measures. Spine.

[21] Landis JR, Koch GG (1997). The measurement of observer agreemen for categorical data. Biometrics.

[22] Giger JT, Barnhart S, Feltner F, Slone M, Lawler MJ, Windsor L (2021). Validating the eHealth Literacy Scale in rural adolescents. J Rural Health.

[23] Tomas CC, Queiros PJ, Ferreira TJ (2014). Analysis of the psychometric properties of the Portuguese version of an eHealth literacy assessment tool. Referência (Coimbra).

[24] Gazibara T, Cakic J, Cakic M, Pekmezovic T, Grgurevic A (2019). eHealth and adolescents in Serbia: psychometric properties of eHeals questionnaire and contributing factors to better online health literacy. Health Promot Int.

[25] Eysenbach G (2020). How to Fight an Infodemic: The Four pillars of infodemic management. J Med Internet Res.

[26] Tangcharoensathien V, Calleja N, Nguyen T, Purnat T, D'Agostino M, Garcia-Saiso S (2020). Framework for managing the COVID-19 infodemic: methods and results of an online, crowdsourced WHO Tecnica Consultation. J Med Internet Res.

[27] Nordheim LV, Gundersen MW, Espehaug B, Guttersrud Ø, Flottorp S (2016). Effects of school-based educational interventions for enhancing adolescents abilities in critical appraisal of health claims: a systematic review. PLoS One.

[28] Pettersen S (2005). Critical thinking in Norwegian upper secondary biology education: the cases of complementary-alternative-medicine and health claims in the media. Nord Stu Sci Educ.

[29] Lee J, Lee EH, Chae D (2021). eHealth literacy instruments: systematic review of measurement properties. J Med Internet Res.

